# Complex Perioperative Decision-Making: Liver Resection in a Patient with Extensive Superior Vena Cava/Right Atrial Thrombus and Superior Vena Cava Syndrome

**DOI:** 10.1155/2016/2106242

**Published:** 2016-01-20

**Authors:** Benjamin Kloesel, Robert W. Lekowski

**Affiliations:** ^1^Department of Anesthesia, Perioperative and Pain Medicine, Boston Children's Hospital, Boston, MA 02115, USA; ^2^Department of Anesthesia, Perioperative and Pain Medicine, Brigham and Women's Hospital, Boston, MA 02115, USA

## Abstract

The perioperative management of patients suffering from extensive superior vena cava (SVC) thrombus complicated by SVC syndrome presents unique challenges. The anesthesiologist needs to be prepared for possible thrombus dislodgement resulting in pulmonary embolism and also has to assess the need for fluid resuscitation given the dangers of massive intravenous fluid application via the upper extremities. We present our perioperative approach in management of a patient scheduled for right hepatectomy who was previously diagnosed with extensive SVC and right atrial (RA) thrombus complicated by SVC syndrome.

## 1. Introduction

Obstruction of the venous inflow tract to the right heart and the presence of an intracardiac thrombus have multiple implications for anesthesiologists that require careful preparation and communication with the involved subspecialties during the perioperative period. Our case presentation will delineate anesthetic considerations, preoperative preparation, and intraoperative management of a patient with central venous catheter- (CVC-) related SVC and RA thrombus complicated by SVC syndrome who presented for resection of multiple liver metastases from a colorectal primary carcinoma.

## 2. Case Presentation

**Figure 1:**
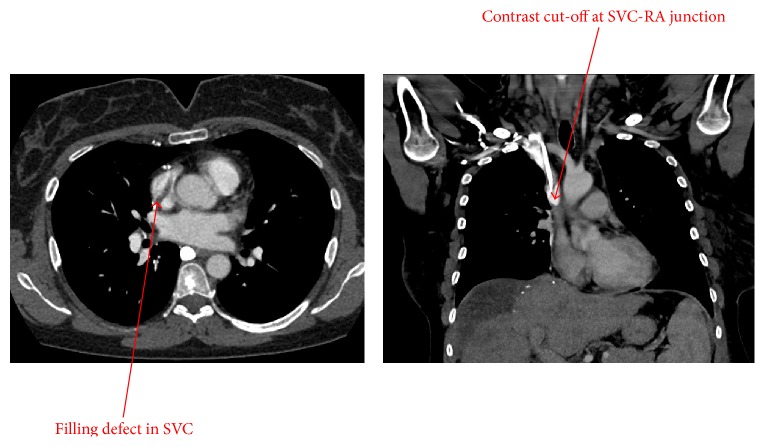
CT angiography of the chest showing a filling defect in the SVC and contrast cut-off at the SVC-RA junction caused by a thrombus.

A 49-year-old female presented to the oncologic surgery service for right hepatectomy and wedge resection of segment 3 of the left lateral liver to remove four right hepatic lobe and one left hepatic lobe colon cancer metastases. Her past medical history was significant for sigmoid adenocarcinoma status after low anterior resection and four cycles of FOLFOX chemotherapy (leucovorin, 5-fluorouracil, and oxaliplatin) administered via a port-a-cath. Her course was complicated by development of a port-a-cath associated thrombus comprised of two parts spanning a total length of 8 cm, with extension from the left brachiocephalic vein along the SVC into the RA and RA appendage (Figure 1). Secondary to the thrombus, the patient developed two episodes of SVC syndrome with facial and neck swelling as well as shortness of breath. The first episode at time of diagnosis was managed with discontinuation of her oral contraceptive pill and intravenous heparin with transition to subcutaneous enoxaparin (1.5 mg/kg once daily), a low-molecular-weight heparin (LMWH), in the outpatient setting. Initially, the port-a-cath was scheduled to be removed, but given concerns from interventional radiology in regard to reinsertion of a CVC in the presence of an extensive SVC thrombus and decrease of thrombus burden with therapeutic anticoagulation, the port-a-cath was left in place. During the second episode, which occurred about three months after initial SVC thrombus diagnosis, the patient was admitted for evaluation of possible catheter-based extraction therapies. On further review of her imaging studies and consideration of the clot appearance and time from initial discovery, the interventional cardiology service felt that, due to clot chronicity, an extraction therapy would not be feasible. The recommendation was made to continue anticoagulation and proceed with surgery after inferior vena cava filter placement. Her surgery was scheduled six months after initial discovery of the port-a-cath associated clot. The patient's anticoagulation management was based on recent guidelines published in 2012 [1]. LMWH was chosen over vitamin K antagonists since clinical trials have shown improved outcomes in patients with solid tumors treated with this regimen [2–4].

A discussion was held with the patient regarding the risks of massive pulmonary embolism and possible therapies. The patient voiced her wish to remain full code and asked for resuscitative efforts to be carried out in the setting of a massive pulmonary embolism. On the day of surgery, a perfusionist and a cardiothoracic surgery team were on stand-by. A perfusion pump was positioned outside of the operating room to allow rapid access to cardiopulmonary bypass capabilities. Arterial access via a 20G catheter was obtained in the right radial artery prior to induction. Anesthesia was induced using a standard induction regimen of midazolam (2 mg) in the preoperative area, followed by fentanyl (100 mcg), propofol (200 mg), and rocuronium (40 mg) via a 20G peripheral intravenous (PIV) catheter in the right upper extremity. No significant lag in medication onset of effect was noted. Bag-mask ventilation was easily achieved. A cuffed 7.0 endotracheal tube was inserted using a MAC 3 blade. Anesthesia was maintained with a volatile agent (sevoflurane) and intermittent fentanyl boluses. After induction, large-bore central venous access was obtained via the right femoral vein under ultrasound guidance to provide means of vasopressor, fluid, and blood product administration. A transesophageal echocardiography (TEE) probe was inserted and images of the right heart thrombus were obtained. The probe was left in place and the thrombus position was periodically checked to confirm the absence of dislodgement. Echocardiography was also used to assess the patient's heart during any signs of hemodynamic alterations. The exam focused on presence of the thrombus, right ventricular systolic function, right ventricular cavity dilation, presence of new regional wall motion abnormalities, occurrence of new tricuspid insufficiency, and position of the ventricular septum. The surgery was carried out in the reverse Trendelenburg position and the patient's eyes and head were monitored every 20 minutes for evidence of swelling. We aimed at limiting intravenous fluids while maintaining adequate intravascular volume and tissue perfusion. Fluid management was guided by stroke-volume variation obtained from a FloTrac/Vigileo^TM^ monitor (Edwards Lifescience Corp., Irvine, CA). A consistent increase of stroke-volume variation above 12% for five minutes was used as a trigger for a 250 cc albumin 5% bolus. The surgery was uneventful and well tolerated by the patient. Procedure time from patient arrival in the operating room to transfer of the patient who is awake to the postoperative anesthesia care unit was 405 minutes. Estimated blood loss was 400 cc. A total of 750 cc of 5% albumin and 1800 cc of Lactated Ringer's solution were given. Intermittent monitoring of the RA thrombus showed no changes and the patient remained hemodynamically stable. After recovery in the postanesthesia care unit, a 20G PIV catheter was inserted in the lower extremity and both femoral CVCs were removed to reduce the risk of clot formation. The patient's enoxaparin was restarted on postoperative day #6 and she was discharged on postoperative day #7.

## 3. Discussion

In daily practice, anesthesiologists are not only required to have a good understanding of the patient's comorbidities and the implications of the upcoming surgery; they also need to be able to anticipate possible difficulties and complications. Along this line, the well-prepared clinician has proactively considered management options for the complications and has made sure that the necessary resources are available to respond quickly and appropriately.

Considerations for liver resection surgery include the possible presence of impaired organ function secondary to the malignant process, metastases, and/or chemotherapy. The liver is a highly vascular organ, and its proximity to large vessels carries the risk for significant intraoperative bleeding that necessitates rapid and adequate volume resuscitation. This stands in stark contrast to the initial goal of keeping the patient slightly hypo- to euvolemic in order to avoid increases in central venous pressure which are associated with hepatic congestion and increased blood loss [5, 6].

The presence of an extensive thrombus in the venous system that extended to the RA, leading to previous episodes of SVC syndrome, added another layer of complexity to this case. The incidence of CVC-associated thrombosis in cancer patients varies, depending on the study, between 0–28% for symptomatic and 27–66% for asymptomatic events. Furthermore, 10–15% of those patients develop pulmonary embolism [7]. While the use of upper extremity intravenous access for delivery of drugs and small volumes of fluids was considered possible (in part due to the chronicity of the condition which induced adequate venous collateralization), large volume resuscitation in the setting of massive blood loss could have triggered another episode of superior vena cava syndrome. Sequelae of this might have included significant airway swelling and raised intracranial pressure from venous congestion. Adequate resuscitation via an upper extremity access point might also have proved inadequate. Consequently, we opted for lower extremity access, which, in turn, also had considerable implications: (a) ideally, we would have aimed for multiple large-bore PIV catheters, but the patient's vascular system did not provide adequate targets; (b) the insertion of a CVC in itself increases the risk for clot formation (hence, the decision to remove the catheters as soon as the immediate period with risk for massive blood loss was over).

Another concern in regard to the extensive thrombus was the possibility of dislodgement and propagation to the pulmonary artery. Pulmonary embolism has been described in numerous case reports of RA thrombus [8–10], with most cases arising from hemodialysis catheters. In our case, the patient was evaluated by cardiology, interventional cardiology, and cardiothoracic surgery. While interventional procedures were deemed unlikely to succeed given the clot chronicity, an open thrombectomy was considered to have an unfavorable risk-benefit ratio. The cardiology service considered the time span from detection of the thrombus to the day of surgery to be sufficient to expect clot organization resulting in a low likelihood of embolism. Nevertheless, for the managing anesthesiologist, the question of “what to do in case the patient suffers a massive pulmonary embolism during the case” needed to be addressed.

The presentation of a perioperative pulmonary embolism covers a large spectrum, reaching from no clinical changes in very small emboli to cardiopulmonary arrest with massive embolization. Pathophysiological changes include V/Q mismatch due to an increase in alveolar dead space resulting in right-left shunting, hypoxia, increased right ventricular (RV) afterload, RV ischemia/infarction, and decrease in cardiac output secondary to decreased left ventricular preload and impaired left ventricular filling from displacement of the interventricular septum to the left [11, 12]. A drop in end-tidal CO2 is related to a decrease in cardiac output. ECG changes can include sinus tachycardia, atrial dysrhythmia, RV strain, right bundle branch block, and SI Q3 T3 pattern (rare) [13]. Transesophageal echocardiography is an excellent tool to diagnose intraoperative pulmonary embolism [14–16]. While direct visualization of thrombus in the pulmonary artery is only sometimes possible (46% of patients in a study by Rosenberger et al. [15]), the echocardiographer can search for evidence of right heart strain such as RV dilation or RV systolic dysfunction [17].

Treatment of pulmonary embolism in the outpatient setting consists of therapeutic anticoagulation with unfractionated or low-molecular-weight heparin followed by transition to warfarin [18]. In the setting of a massive pulmonary embolism leading to hemodynamic instability, thrombolysis with plasminogen-activating fibrinolytic agents [17], catheter-based thrombus reduction via pharmacological and/or mechanical methods [17, 19], or surgical thromboembolectomy [17, 20] can be considered. For sudden cardiovascular collapse, initiation of cardiopulmonary bypass and venoarterial ECMO [21] are considered last resort measures but are limited by their availability and the timeframe to their successful institution.

Thrombus development in the RA has frequently been described in presence of indwelling vascular catheters [10, 22–24] or pacemaker wires [8, 25] and seems to be associated with catheter tip position in the RA as compared to the superior vena cava [26, 27]. A review of six studies by an international working committee identified the following risk factors for CVC-associated thrombosis: CVC tip location above junction between SVC and RA, left-sided CVC insertion, femoral vein access, placement duration of greater than 25 minutes, greater than 1 placement attempt, previous CVC insertion and CVC blockage, and use of triple- (versus double-) lumen CVC and external (versus internal) CVC [7]. Predictors of which atrial thrombi subsequently result in pulmonary embolism are lacking, although there is evidence that this condition is certainly not benign. Kingdon et al. [24] describe a case series composed of 5 patients who developed hemodialysis-catheter associated thrombus. In 3 out of the 5 patients, a pulmonary embolism was documented while another patient suffered a PEA arrest without definitive evidence of pulmonary embolism.

In conclusion, our patient raised multiple considerations for the conduct of safe anesthesia for a liver resection in the setting of SVC syndrome secondary to extensive SVC and RA thrombus. In our report, we delineate our perioperative preparation for possible massive volume loss, vascular access, intraoperative monitoring of thrombus, and an action plan for massive pulmonary embolism.
